# Supplementary material for: Neurophysiological Oscillatory Mechanisms Underlying the Effect of Mirror Visual Feedback-Induced Illusion of Hand Movements on Nociception and Cortical Activation
Source: Brain Sci. 2024 Jul 12;14(7):696. doi: 10.3390/brainsci14070696 (PMC11274372; doi:10.3390/brainsci14070696)
Supplement: Supplementary file 1 [file brainsci-14-00696-s001.zip › brainsci-3078111-supplementary.pdf]

Supplementary Materials

Control analysis

Methods and statistical analysis

Event-related potentials (ERPs) in response to the auditory cues and electrical stimuli were extracted from the Cz electrode. Successively, the waveforms resulting from painful and non-painful blocks were averaged across the subjects for each condition and paired samples t-tests were used for statistical comparison. The ERPs considered were the N1-P2 (auditory), P2-N2 complex (electrical stimulus), and P3 (attentional). Furthermore, the randomness of the painful and non-painful epochs was tested by performing the RUNS test for each subject and condition. Finally, NRS values were compared in order to control differences in the subjective perception of the stimulus intensity for each condition. All the statistical analyses were performed using IBM SPSS Statistics 27 (IBM, New York, USA).

Results

The ERP waveforms are plotted in Fig. S1. Results indicate that the negative peaks (N1 and N2) present higher amplitude in the painful (red line) than non-painful (black line) blocks. This was true at a statistical level for the N1 in the UM+ condition ($p < 0.05$) as well as for the N2 in the UM- condition ($p < 0.05$). Moreover, the P2 peak showed higher amplitude in the painful than non-painful blocks in the UM+ condition ($p < 0.05$). Interestingly, no differences were observed for the attentional P3 ERP. No statistically significant differences were found in the latency measures. All the mean and standard error values are reported in Table S1.

The RUNS test's results for the experimental condition (UM+) showed no significant differences (average number of Runs = 6.8; SD = ± 1.6; $p > 0.05$) (Figure S2), indicating that the order of the blocks in which the subjects perceived the electrical stimuli as painful or non-painful presents a random distribution (i.e., the variability in the individual perceived intensity was not due to habituation or sensitization effects).

Finally, paired samples t-tests showed that NRS values relative to painful blocks are significantly higher than values in the non-painful blocks for each condition ($p < 0.001$ for all comparisons) (Figure S3).

The time-frequency analysis (Fig. S4) confirms a prominent reduction of the alpha frequency before and after the auditory cue (Fig. S4, dotted purple line) contralateral to the moving hand (C3) for each condition. We can also observe a power increase in the delta (1-4 Hz) and theta (4-7 Hz) activity in the UM- (Fig. S4-a) and UM+ (Fig. S4-c) conditions during the movement execution. These oscillations over the primary motor cortex are shown to synchronize with the timing of informative cues employed to initiate movement planning (Saleh et al., 2010; Körmendi et al., 2021) and cognitive control (Pellegrino et al., 2018; Schramm et al., 2019). A strong power increase in the beta (about 12-30 Hz) and gamma (>30 Hz) frequency can be observed in the UM+ condition (Fig. S4-c) after movement execution. This may reflect beta the well-known post-movement beta rebound (Heinrichs-Graham et al., 2017).

Figure S1

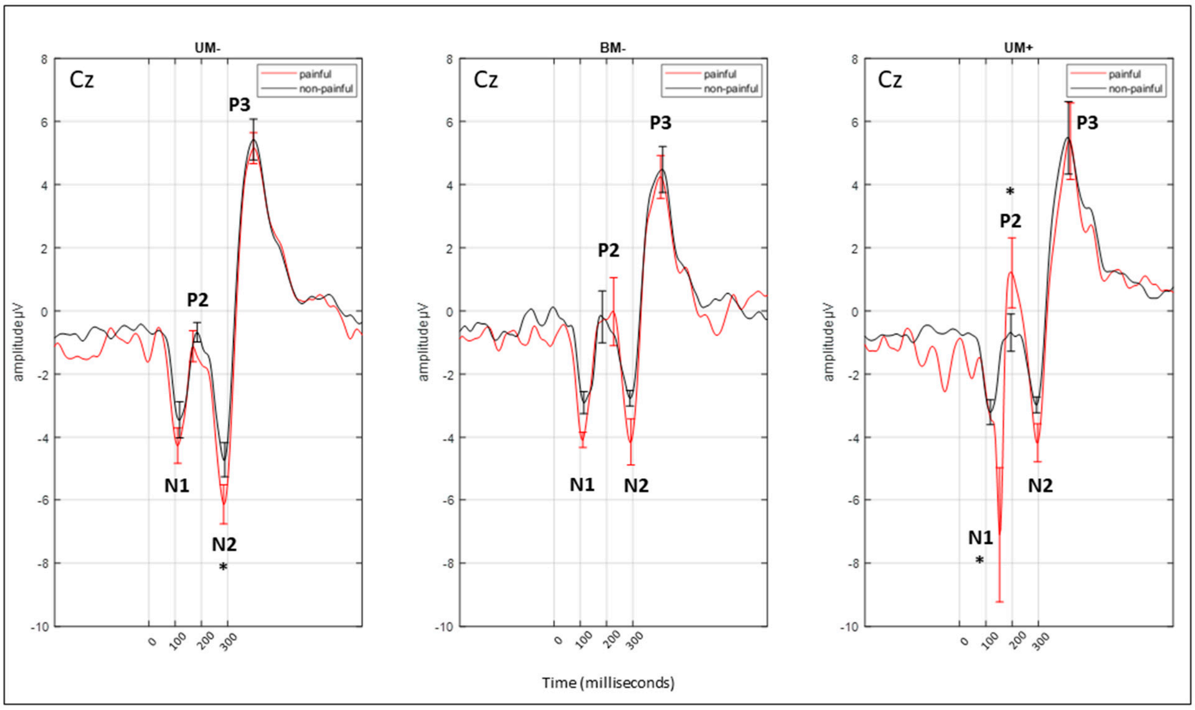

The figure illustrates the mean event-related potentials (ERPs) generated in response to auditory cues and electrical sensory stimuli at the Cz electrode. The differences between painful (red line) and non-painful (black line) blocks are represented for each condition (UM–, BM–, and UM+). Statistical analysis (t-tests) showed significant differences in the N2 peak for the UM- condition as well as in the N1 and P2 peaks for the experimental UM+ condition (*p < 0.05).

Table S1

	N1		***P2***		***N2***		***P3***	
	Pain	*No pain*	*Pain*	*No pain*	*Pain*	*No pain*	*Pain*	*No pain*
	Mean ± SE		*Mean ± SE*		*Mean ± SE*		*Mean ± SE*	
UM–	*-4.26 ± 0.56*	*-3.91 ± 0.57*	*-0.99 ± 0.49*	*-0.61 ± 0.31*	*-6.13 ± 0.62*	*-4.77 ± 0.54*	*5.37 ± 0.49*	*5.65 ± 0.65*
BM–	*-4.07 ± 0.24*	*-2.96 ± 0.35*	*-0.04 ± 1.08*	*-0.24 ± 0.82*	*-4.21 ± 0.73*	*-2.84 ± 0.25*	*4.31 ± 0.68*	*4.49 ± 0.73*
UM+	*-7.26 ± 2.13*	*-3.39 ± 0.39*	*1.61 ± 1.11*	*-0.67 ± 0.59*	*-4.17 ± 0.60*	*-3.26 ± 0.25*	*5.63 ± 1.21*	*5.69 ± 1.15*

The table shows the mean and standard error values (expressed in mV) for each condition (UM–, BM–, and UM+) for both the painful and non-painful blocks. All the event-related potentials (ERPs: N1, P2, N2, and P3) are reported. The significant differences are shown in Figure S1.

Figure S2

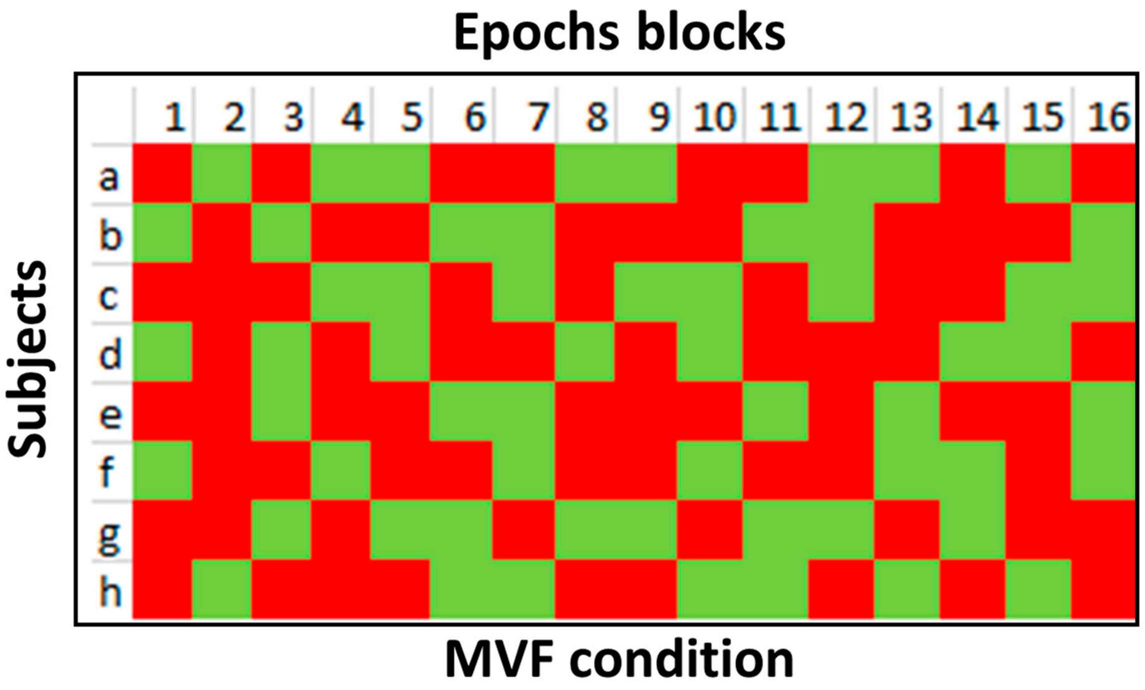

The graph shows the distribution of the blocks where each subject (Y-axis) perceived the stimuli as painful (NRS > 5, red blocks) or non-painful (NRS < 5, green blocks) in the experimental Unilateral Mirror (UM+) condition. Each block consisted of 5 trials. Therefore, a total of 16 blocks are shown on the X-axis. The RUNS test showed that the red and green blocks are randomly distributed for each subject ($p < 0.05$).

Figure S3

a

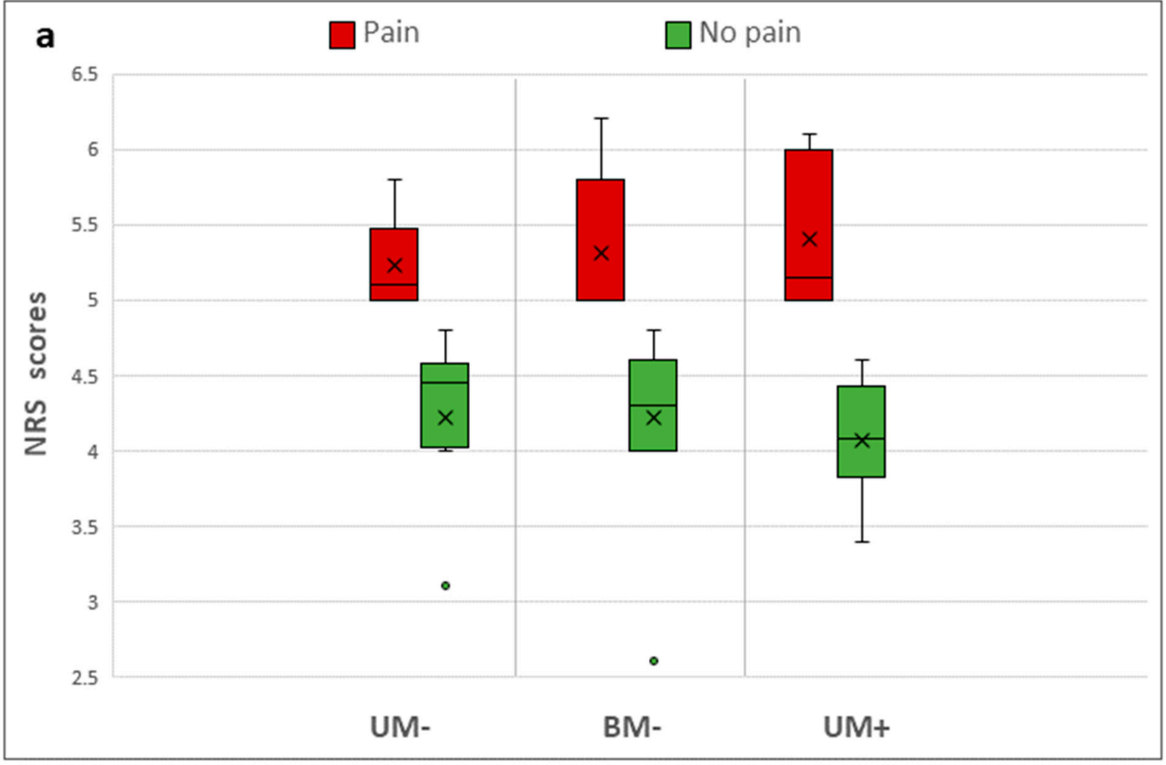

b

Mean ± SE	UM-	BM-	UM+
Pain	5.23 ± 0.08	5.31 ± 0.14	5.40 ± 0.17
No pain	4.22 ± 0.16	4.21 ± 0.18	4.07 ± 0.13

The boxplot displays the extremes, the upper and lower quartiles, the median (line), and the mean (x) of the Numerical Rating Scale (NRS) values provided by the subjects during each condition (a). The ratings were separated into painful (NRS > 5, red) and non-painful (NRS < 5, green). Paired samples t-tests controlled that the painful and non-painful ratings differed significantly in each condition ($p < 0.001$). The table below (b) shows the mean and the standard error (SE) for each condition and perceived intensity.

Figure S4

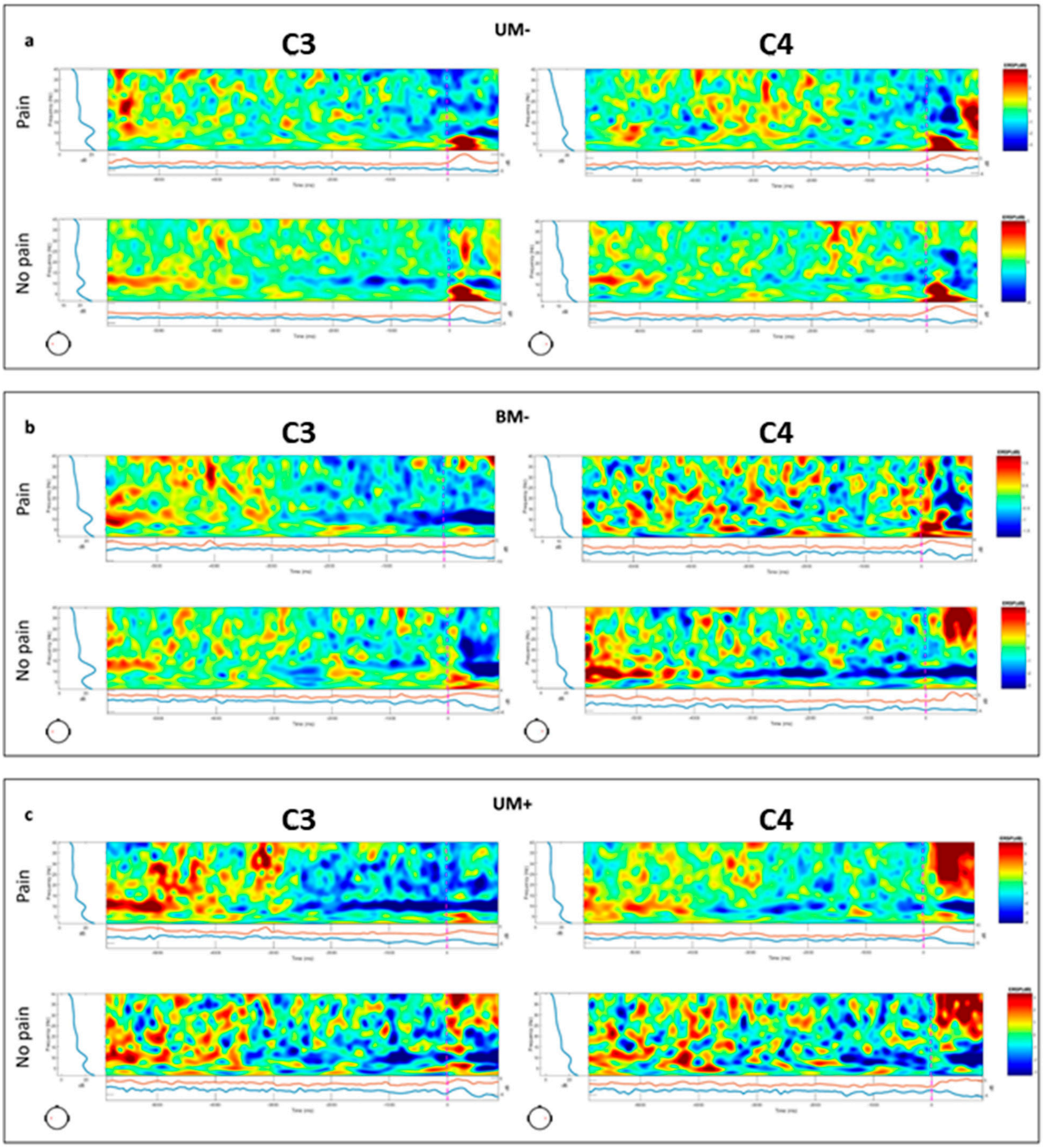

The figure shows the across-subjects mean event-related spectral power (ERSP) for the pain and no-pain blocks and each condition (*UM–, BM–*, and *UM+*) at each frequency (from 0 to 40 Hz). The entire epochs (i.e., from 7 s before the auditory cue to 1 s after the cue) were averaged for each subject and condition. Only the electrodes overlying the left and right sensory-motor cortex (i.e., C3 and C4) were reported. The left panels show the baseline mean power spectrum, whereas the lower panels indicate the low and high mean values (expressed in dB) at each time in the epoch. In the figure, the dark blue colour

represents a power reduction in the given frequency, whereas the dark red colour represents a power increase in the frequency.

[1–5]

References

1. Heinrichs-Graham, E.; Kurz, M.J.; Gehringer, J.E.; Wilson, T.W. The Functional Role of Post-Movement Beta Oscillations in Motor Termination. *Brain Struct. Funct.* **2017**, *222*, 3075–3086, doi:10.1007/s00429-017-1387-1.
2. Körmendi, J.; Ferentzi, E.; Weiss, B.; Nagy, Z. Topography of Movement-Related Delta and Theta Brain Oscillations. *Brain Topogr.* **2021**, *34*, 608–617, doi:10.1007/s10548-021-00854-0.
3. Pellegrino, G.; Tomasevic, L.; Herz, D.M.; Larsen, K.M.; Siebner, H.R. Theta Activity in the Left Dorsal Premotor Cortex During Action Re-Evaluation and Motor Reprogramming. *Front. Hum. Neurosci.* **2018**, *12*, 364, doi:10.3389/fnhum.2018.00364.
4. Saleh, M.; Reimer, J.; Penn, R.; Ojakangas, C.L.; Hatsopoulos, N.G. Fast and Slow Oscillations in Human Primary Motor Cortex Predict Oncoming Behaviorally Relevant Cues. *Neuron* **2010**, *65*, 461–471, doi:10.1016/j.neuron.2010.02.001.
5. Schramm, S.; Albers, L.; Ille, S.; Schröder, A.; Meyer, B.; Sollmann, N.; Krieg, S.M. Navigated Transcranial Magnetic Stimulation of the Supplementary Motor Cortex Disrupts Fine Motor Skills in Healthy Adults. *Sci. Rep.* **2019**, *9*, 17744, doi:10.1038/s41598-019-54302-y.
